# Supplementary material for: Time‑resolved multi-omic analysis of paclitaxel exposure in human iPSC‑derived sensory neurons unveils mechanisms of chemotherapy‑induced peripheral neuropathy
Source: Cell Death Dis. 2026 Feb 10;17(1):211. doi: 10.1038/s41419-026-08445-2 (PMC12921266; doi:10.1038/s41419-026-08445-2)
Supplement: Supplementary file 15 — Dataset 1. [file 41419_2026_8445_MOESM15_ESM.zip › CDD_Open_Data/04_RNA_Seq/Timeline_panel_plots_etc_2023-01-23_1609.zip!/Panel_Plots_etc/Evidence_Plots/post5d/evidence_plot_standard contrasts_msigdb_reactome_5d_PTX_vs_DMSO_ID6_M13748_REACTOME_GLUCONEOGENESIS.pdf]

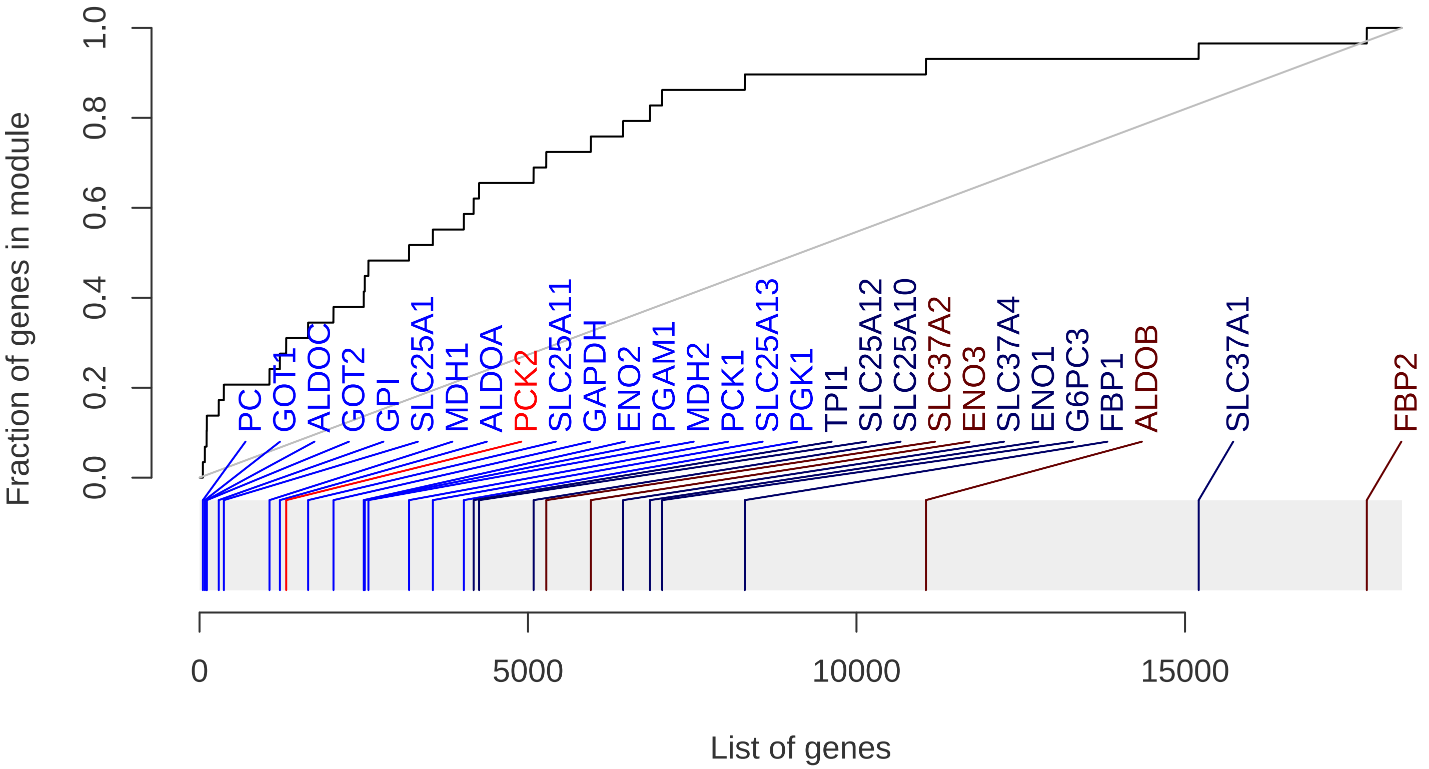

Fraction of genes in module
0.0
0.2
0.4
0.6
0.8
1.0
PC
GOT1
ALDOC
GOT2
GPI
SLC25A1
MDH1
ALDOA
PCK2
SLC25A11
GAPDH
ENO2
PGAM1
MDH2
PCK1
SLC25A13
PGK1
TPI1
SLC25A12
SLC25A10
SLC37A2
ENO3
SLC37A4
ENO1
G6PC3
FBP1
ALDOB
SLC37A1
FBP2
0
5000
10000
15000
List of genes
